# The Cinderella tree, *Quillaja saponaria* – A soap story

**DOI:** 10.1002/ppp3.70108

**Published:** 2025-11-25

**Authors:** Anne Osbourn

**Affiliations:** Department of Biochemistry and Metabolism, John Innes Centre, Norwich Research Park, Norwich, NR4 7UH, UK

**Keywords:** Botany, Chile, engineering biology, immunology, QS saponins, QS-21, *Quillaja saponaria*, soapbark, vaccine adjuvants

## Abstract

The Chilean soapbark tree, *Quillaja saponaria* Molina, grows in the central part of Chile. As its name suggests, the tree is a natural source of soap. Indeed, the name *Quillaja* is derived from the indigenous Chilean word ‘küllay’, which means ‘soap’. Soapbark is not the only plant to produce natural soaps. Other examples include the perennial soapwort (*Saponaria officinalis*), which grows in Europe and has historically been used as a source of gentle soap for washing delicate fabrics, including allegedly the Turin shroud; and *Sapindus mukorossi* (soapberry or soapnut), which grows in temperate and tropical regions of the world. The soapy properties of these plants are due to their production of glycosylated compounds known as saponins. In fact, saponins are found across the Plant Kingdom, not just in species with ‘soap’ in their names, and are one of the largest groups of plant natural products. The major saponin produced by *Q. saponaria*, which is known as QS-21, is a potent immunostimulant and has been approved for use in human vaccines. QS-21, which is the first naturally occurring plant glycoside to be used as an adjuvant, is a highly complex molecule that is produced only by *Quillaja*. This review covers the history of the soapbark tree, the discovery of QS-21 and the potential for making the next generation of saponin vaccine adjuvants using engineering biology approaches.

## 1 Introduction

**Figure 1:**
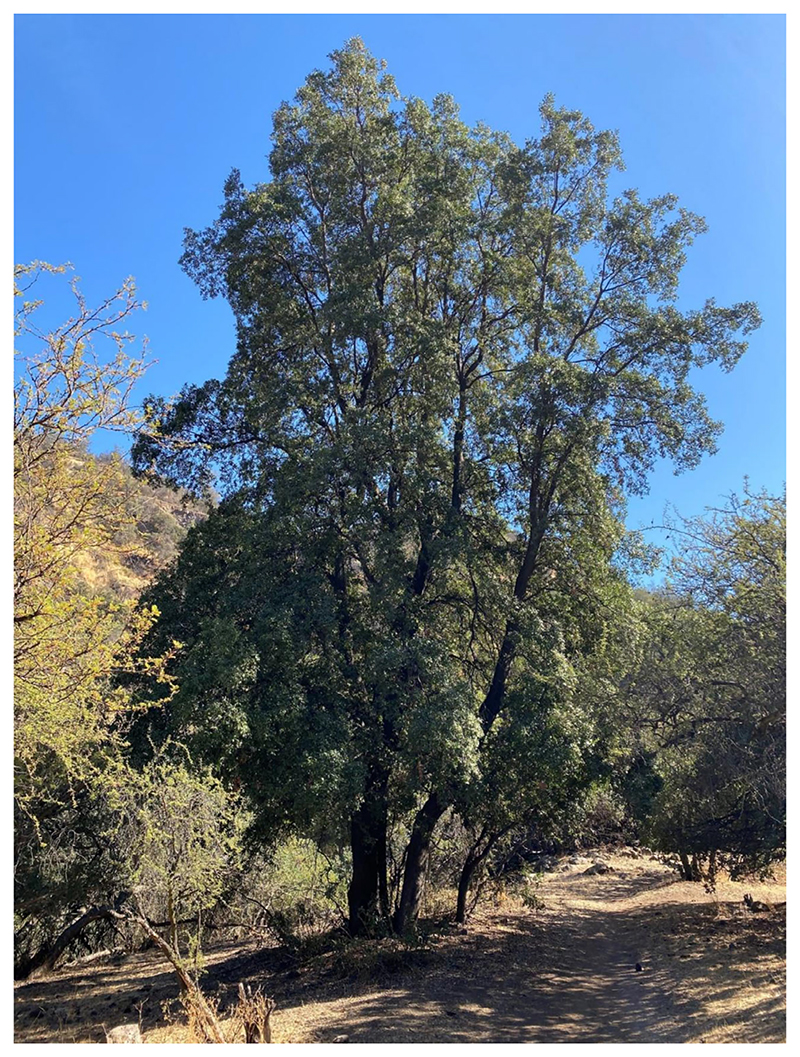
A wild quillay tree growing in central Chile. Image provided by Cristobal Uauy.

**Figure 2:**
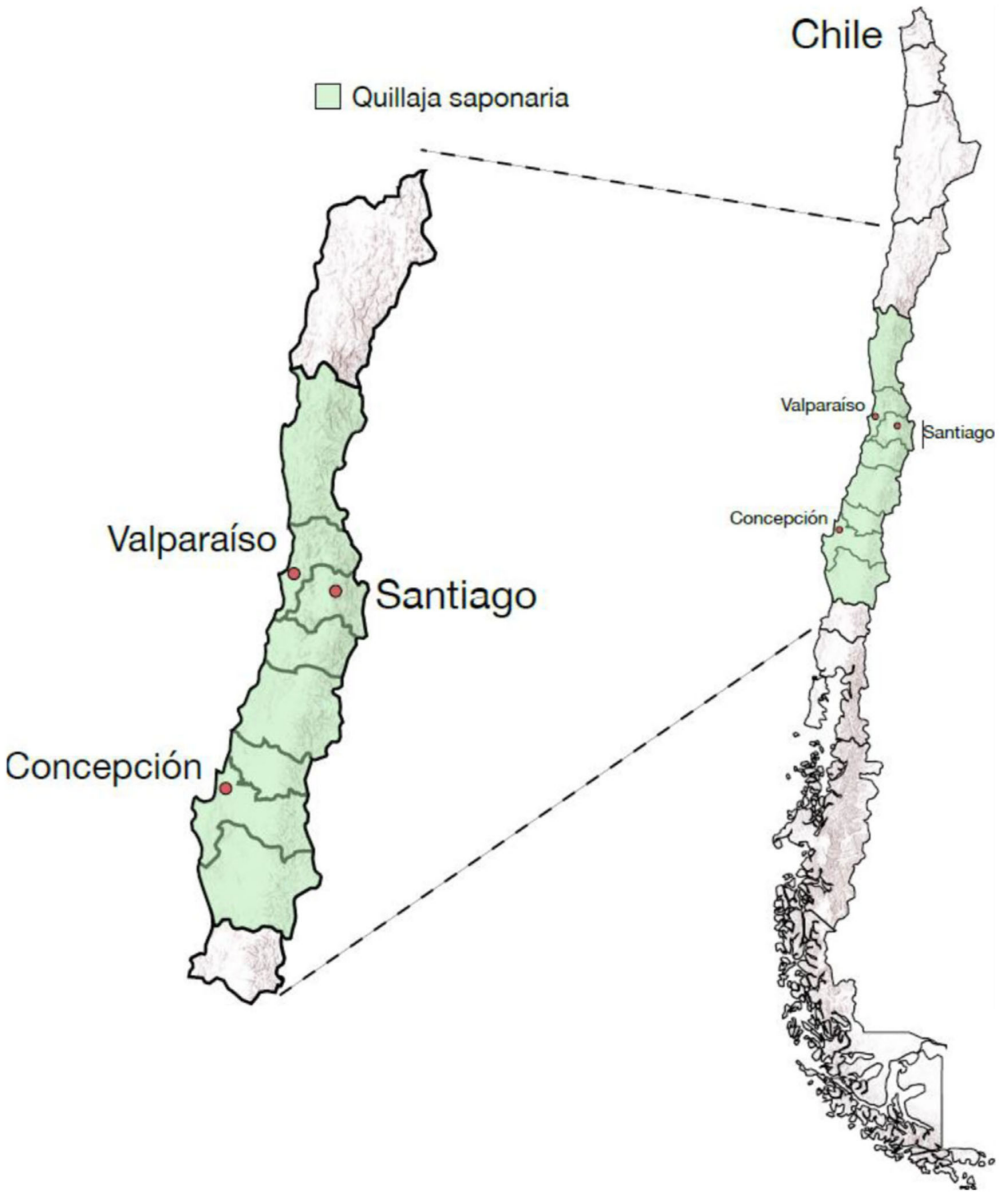
Map showing the distribution of quillay in Chile. The distribution of the quillay tree is taken from the Kew Gardens Plants of the World Online website (Quillaja saponaria Molina | Plants of the World Online | Kew Science). The Scalable Vector Graphics image file for Chile and administrative regions is from: https://mapsvg.com/maps/chile, and the elevation information from the Environmental Systems Research Institute: (https://www.esri.com/arcgis-blog/products/arcgis-online/announcements/topographic-with-contours-multisource-vector-tile-layers/). This image was generated by Bernardo Pollak.

The soapbark tree *Q. saponaria* Molina, or quillay, grows in central Chile (Figure 1). Its name originates from ‘küllay’, a word used by the local indigenous people (the Mapuches) meaning ‘soap’ (https://wold.clld.org/vocabulary/41). The foaming properties of quillay bark have a long traditional use as soaps and shampoos. The native distribution of *Q. saponaria* is shown in Figure 2. A related species, *Quillaja brasiliensis* (A. St.-Hil. & Tul.) Mart, grows in southern Brazil, northern Uruguay, northeastern Argentina and eastern Paraguay (Luebert, 2013). The story of the Chilean soapbark tree is intriguing. Unlike the Canelo tree *Drimys winteri* J.R. Forst. & G. Forst., another South American tree that also grows in Chile, it does not appear to have any religious or ceremonial connotations (Frézier, 1717; Molina, 1782). It was largely overlooked by the early European plant collectors, who were distracted by the gaudier botanical offerings that they found when travelling through South America. In the eighteenth and nineteenth centuries, the bark was exported for its soap and shampoo properties, while the wood was hard and useful for making stirrups. However, the soapbark tree was not centre stage in terms of its commercial potential. It did not have the value of the much sought after quinine tree (*Cinchona* spp.), prized because of its anti-malarial properties. The tables turned when it was found that *Q. saponaria* bark extract has potent immunostimulant activity when added to animal vaccines. This ultimately led to the discovery of QS-21, a triterpenoid glycoside (saponin) purified from crude bark extract that was approved for use as an adjuvant in human vaccines in 2017.

QS-21 is currently a critical component of commercial vaccines for malaria, shingles and respiratory syncytial virus and also a further ~ 40 other vaccines under development. For these reasons, it costs in excess of $100,000/g. It is mainly sourced by extraction from the bark of wild *Q. saponaria* trees. Demand for the compound has led to growing concern about the sustainable supply of QS-21 as more vaccines are approved for human use (Schlotterbeck et al., 2015). For example, the two approved malaria vaccines (the AS01-containing vaccine Mosquirix [RTS,S] from GSK and R21, a Matrix-M-containing vaccine developed by the Jenner Institute) both contain QS-based adjuvants. It is estimated that for these alone around 200 million doses of vaccine per year will be needed to protect children in malarious parts of Africa (Honigsbaum, 2024).

This review highlights the story of the soapbark tree – its ‘discovery’ by the early European plant collectors who first went to Chile in the 1700s; the botanical reporting and naming of the tree; and its commercial significance, from export of bark for use as soaps and shampoos to the development of purified bark extract components as vaccine adjuvants. From this narrative, it will become evident that soapbark was largely overlooked from both a botanical and a commercial perspective until the late twentieth century – hence the title of this review: ‘The Cinderella tree.’

## 2 The Plant Collectors: The ‘Discovery’ Of The Quillay Tree By The Europeans

The early European plant collectors in Chile came from France and Spain. Chile was occupied by the Spanish Empire from 1540 to 1818. However, following the accession of the French Prince, Philip of Anjou (later to be known as King Philip V of Spain), to the Spanish throne in 1700, the Spanish colonies also became more accessible to French scientists.

French explorer Louis Feuillée was the first of the European plant collectors to visit Chile, arriving in Concepción in January 1708. He travelled around the country for a month, visiting Valparaíso and its surrounds, and also went on to Peru. During his travels, he collected botanical specimens and made watercolour illustrations of plants before returning to France in 1711. His observations were published in three volumes and included engravings based on his paintings (Feuillée, 1714). Feuillée mentions the Concepción strawberry *Fragaria chiloensis* (L.) Mill. in his writings: ‘Several fruits, like pears, apples, strawberries, etc., were ripe. For dessert, we were served some strawberries of marvellous taste, whose size equalled that of our largest nuts. Their colour is a pale white. They are prepared in the same manner as we fix them in Europe, and, although they have neither the colour nor the taste of ours, they do not lack excellence.’ He did not include a specimen of this strawberry in the botanical collection that he brought back to France. That was done by Amédée-Francois Frézier, who visited Chile a few months after Feuillée’s return. Although Feuillée travelled through the central region of Chile, he did not mention quillay in his publications or illustrate it.

**Figure 3:**
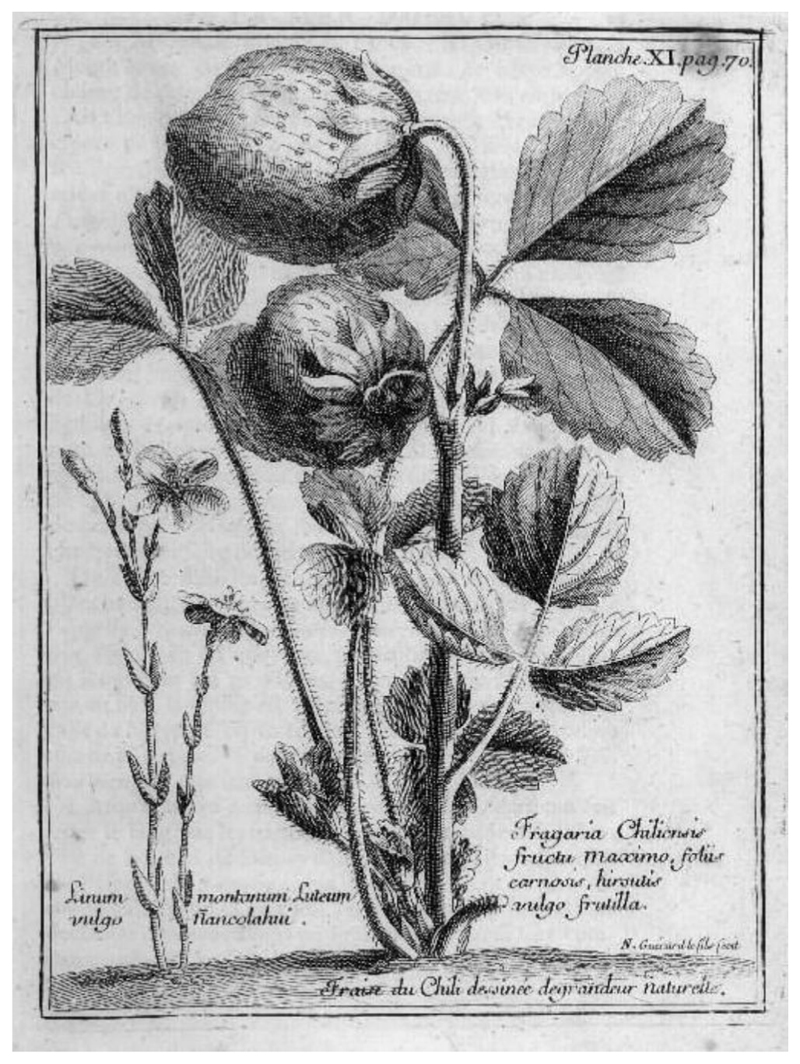
Botanical illustration of the Chilean strawberry, *Fragaria chiloensis*. From Frézier (1717).

Frézier was a French military engineer, mathematician, spy, explorer and also a plant collector. As a lieutenant-colonel of the French Army Intelligence Corps he was dispatched to South America to carry out reconnaissance work, which included taking exact plans of key ports and fortresses along the coast. He left France on January 7th^,^ 1712 on an armed merchant ship (the *St. Joseph*), which travelled around Cape Horn and arrived in Concepción around five months later (on June 16, 1712). Masquerading as a trader, he was able to visit the fortifications as a tourist. He ingratiated himself with the Spanish Governors, and while based in Concepción he sketched maps of the ports showing the best approaches for attack, ammunition storage sites and routes of escape, estimated the strengths of the Spanish colonial governments, the living conditions for Indigenous people and examined the Spanish gold and silver mines. He also reported on the operations of the Church, the physical geography and the flora and fauna of the area, as well as its agricultural products. He is best known for introducing the Chilean strawberry species *F. chiloensis* into Europe (Figure 3). Frézier left Concepción on February 19th^,^ 1714, reaching Marseilles on August 17th. His account of his travels was published in Paris in 1716, and subsequently in England (in 1717) as *A Voyage to the South-Sea, And along the Coasts of Chili and Peru, In the Years 1712, 1713 and 1714* (Frézier, 1717). His observations on the Chilian flora included this note on the quillay tree: ‘The *Quillay* is a Tree, the Leaf whereof somewhat resembles that of the green Oak; its Bark ferments in Water like Soap, and is better for washing of Woollen Cloaths, but not for Linnen, which it makes yellow. All the *Indians* make use of it for washing their Hair, and to cleanse their Heads instead of Combs; it is thought to be that which makes their Hair black.’ He did not, however, include an illustration of the tree.

Quillay was first given its formal binomial name – the species name *Q. saponaria* – by the Chilean-born Jesuit, Juan Ignacio Molina. Like many other Jesuits, Molina was exiled from Chile by the Spanish monarchy. In the 1770s, Molina wrote a natural and social history of Chile while living in Italy. This book, *Saggio*, was first published in 1782 (Molina, 1782). In this, he named quillay *Q. saponaria* Molina according to the taxonomic principles of Linnaeus. Molina was the first Chilean-born naturalist to use Linnaeus’s system.

**Figure 4:**
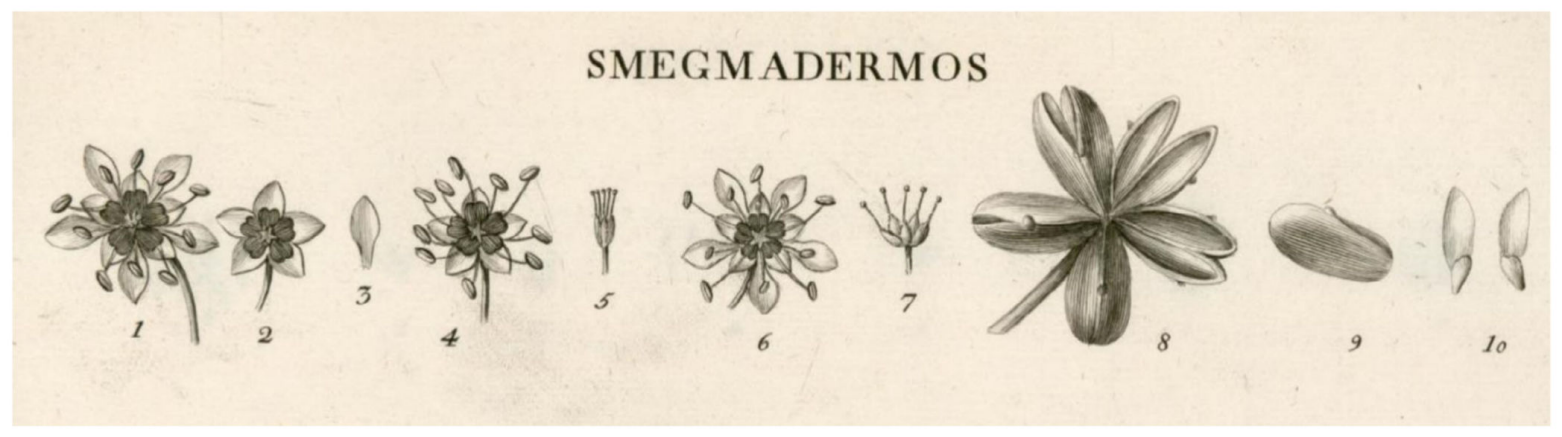
The first known botanical illustration of *Quillaja saponaria* (synonym *Smegmadermos*). The image is from Ruiz and Pavón (1794). The authors assigned quillay to the genus Smegadermos (which means ‘sebacous bark’) because of its ability to serve in place of soap. The image, which was produced by an unidentified member of the expedition team, shows details of the flowers, capsules and seeds, but not the overall architecture of the plant. The details are labelled from left to right as follows: 1. Flower, male hermaphrodite; 2. Chalice showing the disc; 3. Petal; 4. Calyx with stamens; 5. Pistils; 6. Female hermaphrodite flower; 7. Pistils; 8. Capsule closed and open; 9. Capsule seen from the side; 10. Seeds.

Spanish botanists Hipólito Ruiz and José Pavón spent the years 1782–83 studying the Chilean flora, mainly around Concepción. They had several botanical texts with them, including three volumes of the Journal des Observations by Feuillée and various texts by Linnaeus (Burdick & Toledo, 2021; Feuillée, 1714). They were accompanied by the French naturalist Joseph Dombey. Dombey collected dried samples of *Smegadermos emarginata* Ruiz et Pav. which he sent to the Muséum national d’Histoire naturelle in Paris, while in 1782 the Royal Botanical Garden of Madrid received a packet containing ~35 seed capsules collected by Ruiz and Pavón in Concepción, labelled as *Smegmadermos quillaia* (*S. emarginata* Ruiz & Pav. and *S. quillaia* are both synonyms for *Q. saponaria*) (Burdick & Toledo, 2021). Dombey, Ruiz and Pavón were amongst the first to transport quillay to Europe. In May 1783, the team moved to Santiago where they spent the winter advancing drawings and organising a ‘drawer’ of materials to send to Spain. Their initial attempts to send drawings and live plants back to Spain were thwarted by the sinking of the ship that they used to transport them, the *San Pedro de Alcántara*, off the coast of Portugal in 1784. Despite this setback, in 1788 Ruiz brought a second shipment back to Spain with 29 drawers of dried plants, 173 living plants from Peru and Chile, 589 drawings and 2000 botanical descriptions. These drawings included an image showing the flower and seed structures of *Q. saponaria* (Figure 4), possibly the first botanical illustration of the species to reach Europe (Ruiz & Pavón, 1794), although notably no overall image of the tree or illustrations of its leaves are included. At that time, it was challenging to transport living plant material across the oceans by ship because plants need fresh water and protection from the elements. This problem would subsequently be overcome by the development of the Wardian case, a sealed glass container that provided a controlled environment, allowing plants to receive sunlight, protecting them from salt water and feeding them a supply of condensed water (Ward, 1842).

Darwin, on his journey down from the Cordillera de los Andes, went straight through the region where quillay grows, but was clearly unimpressed, as is evident from his diary entry for that day: ‘*April 6*^*th*^ *1835*. – In the morning we found some thief had stolen one of our mules, and the bell of the madrina. We therefore rode only two or three miles down the valley, and staid there the ensuing day in hopes of recovering the mule, which the arriero thought had been hidden in some ravine. The scenery in this part had assumed a Chilian character: the lower sides of the mountains, dotted over with the pale evergreen Quillay tree, and with the great chandelier-like cactus, are certainly more to be admired than the bare eastern valleys; but I cannot quite agree with the admiration expressed by some travellers. The extreme pleasure, I suspect, is chiefly owing to the prospect of a good fire and of a good supper, after escaping from the cold regions above: and I am sure I most heartily participated in these feelings’ (Darwin, 1839). Darwin instead was attracted to the orange-flowered *Berberis darwinii* Hook. (Darwin’s barberry), which has become an invasive species following introduction into other parts of the world.

It is noteworthy that the European plant collectors were drawn to the more flamboyant Chilean plant species such as the showy red/pink Chilean bellflower (*Lapageria rosea* Ruiz & Pav.; now the Chilean National Flower), the tall yellow-spiked bromeliad puyas (*Puya chilensis* Molina), the Chile pine *(Araucaria Araucana* Molina), K. Kock (also known as the monkey-puzzle tree) and *B. darwinii*, as well as, of course, the luscious Chilean strawberry *F. chiloensis*. The more discrete quillay tree tended to go largely unnoticed despite its magnificent tall stature and beautiful star-like flowers, the latter appearing in the Chilean summer, between December and February.

## 3 The Taxonomy Of *Quillaja*

**Figure 5:**
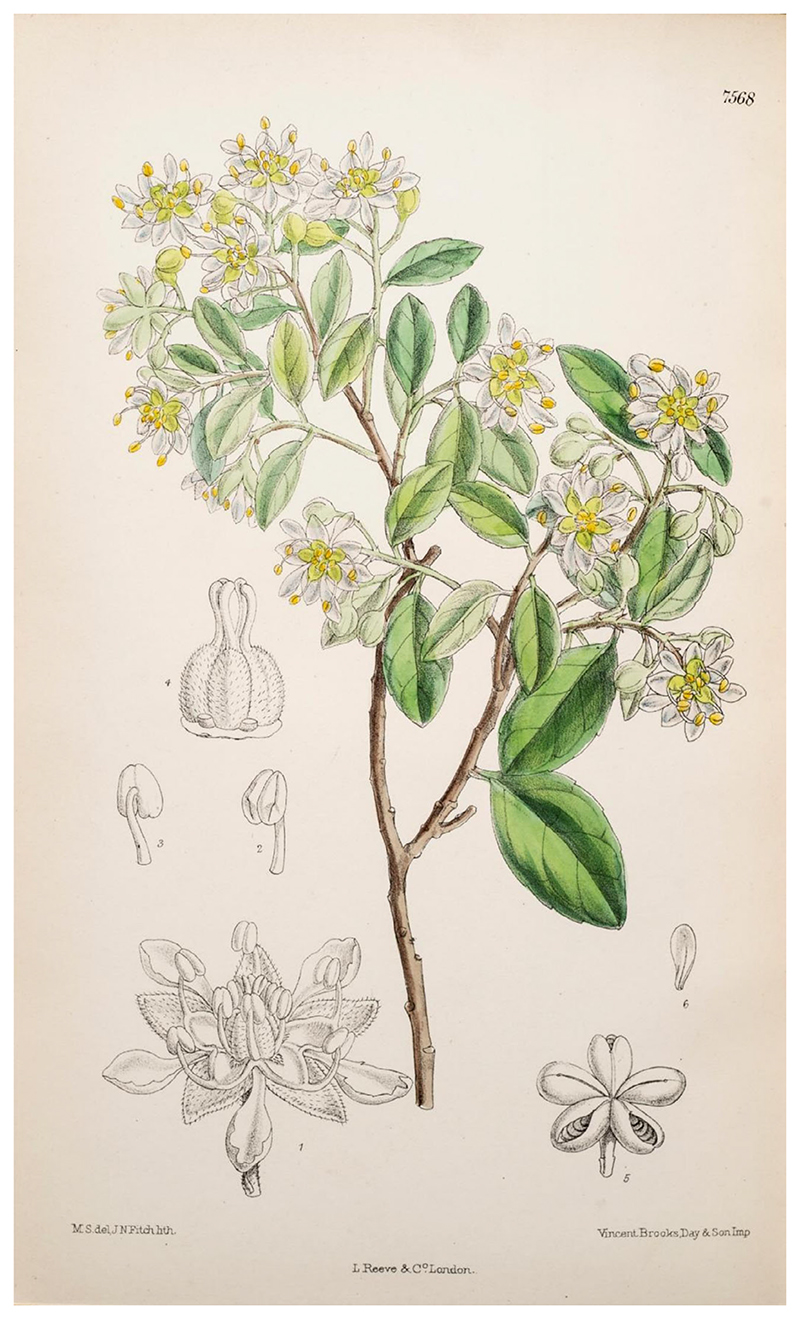
Botanical illustration of *Quillaja saponaria* from Hooker (1897). The artist and lithographer was J.N. Fitch. The illustration was made from a specimen sent by Sir Thomas Hanbury, F.L.S., which flowered in his botanical garden La Mortola, Ventigmiglis, Italy in February 1987.

A description of *Q. saponaria* by Joseph Dalton Hooker in *Curtis’s Botanical Magazine* (Hooker, 1897) reads as follows: ‘– A small tree, thirty to forty feet high, sparingly branched, with ashy bark; branchlets slender, glabrous. *Leaves* one and a quarter to two inches long, very shortly petioled, elliptic or oblong, tip obtuse or rounded, margin entire or undulate; stipules two, small. *Flowers* about two-thirds of an inch broad, few together in a small panicle, greenish yellow, shortly pedicelled, the terminal in the panicle fertile, the lateral male. *Calyx-tube* short; lobes five, ovate, valvate. *Petals* five, small, spathulate. *Disk* fleshy, occupying the base of the calyx and projecting five lobes which are adnate to the surfaces of the calyx-lobes. *Stamens* 10, five opposite the petals inserted in the bottom of the disk, and five on the lobes of the disk; filaments subulate, anthers small. *Carpels* five, cohering by their bases, tormentose, many-ovuled; styles short, stigmas, dilated. *Fruit* of five obovoid coriaceous, tormentose, stellately spreading follicles, seated on the persistent withered calyx.’ Hooker’s article was accompanied by a botanical illustration of *Q. saponaria* by artist Matilda Smith and lithographer J.N. Fitch (Plate 7,568; Hooker, 1897) (Figure 5).

At that time, the species was classified as being in the rose family (Rosaceae; order Rosales), which also includes apples and pears, due to the pentamerous arrangement of the flowers (Burdick & Toledo, 2021). However, *Quillaja* has a number of features that are atypical for the Rosaceae, including clawed petals, two types of stamens, lobed ovaries and winged seeds (Ritter, 2011). Based on these botanical anomalies and on molecular phylogenetic analysis (Bello et al., 2008; Morgan et al., 1994; Savolainen et al., 2000), the genus was subsequently removed from the Rosales order and placed into its own family (Quillajaceae) within the order Fabales (Burdick & Toledo, 2021; Luebert, 2013). Thus, it was not only overlooked by many of the European plant collectors but also ousted from the established plant taxonomic framework of the time.

## 4 Commerce In The Eighteenth and Nineteenth Centuries

**Figure 6:**
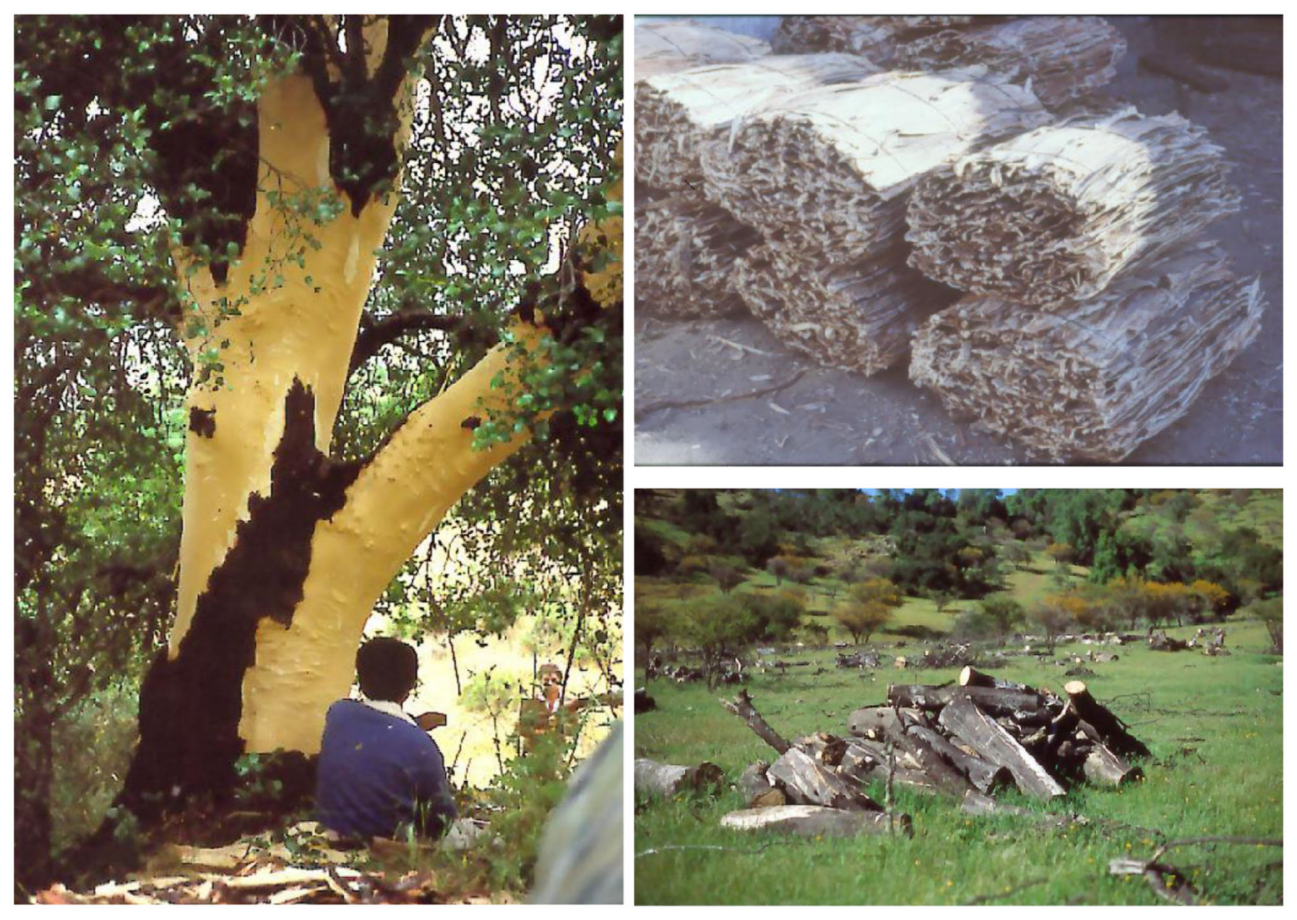
Stripping bark from wild quillay trees. Image provided by Ricardo San Martin.

The discovery of ‘new’ plant species from around the world by European naturalists in the eighteenth and nineteenth centuries was not driven purely by botanical interest, but by the ambitions of countries such as France, Spain and the UK to acquire and describe unknown botanical specimens that had relevance to medicine and commerce (Burdick & Toledo, 2021). Joseph Hooker noted of *Q. saponaria* in a 1904 bulletin (Hooker, 1904) that: ‘Its wood, though not procurable of any great size, is valued for its hardness, and is chiefly used for props in mines, and for making stirrups. Of greater account is its bark, which, when pulverized in water, foams like soap, and is used as an efficacious substitute for that article, as also for dressing the hair. Both Chilians and Araucarian Indians attribute the luxuriance of this ornament of their persons to its use. There is considerable import of the bark into England; and it appears annually in the trade lists, the wholesale price being 6 d per pound, and pulverized 1 s. A detergent hair-wash is prepared from it, and has been extensively used to produce a head on stale beer’ (Figure 6).

While the primary use of *Q. saponaria* does not appear to have been medicinal, Hooker’s article (Hooker, 1904) notes that quillaia bark had been included in the U.S. Pharmacopoeia; also that it was listed as officinal in the French Codex under the name *Bois de Panama* (in reference to the historical trading route through which the bark came to Europe). The tincture made from it was chiefly employed as an emulsifying agent for the preparation of various balms and oils, rather than as a medicinal compound per se. However, Hooker also commented that *Q. saponaria* bark had been proposed by Dr. R. Kobert as a more palatable and cheaper substitute for senega snakeroot, which was at the time used for the treatment of earache, toothache and sore throats, and as an expectorant for croup and colds. Dr. Kobert had reported that patients tolerated quillaia better than senega, that it rarely caused vomiting or diarrhoea, was readily taken by children, and had superior expectorant activity. The preparation used by Dr. Kobert was a decoction made from five parts of the bark to 200 of water, of which the dose was a teaspoonful for children and a tablespoonful for adults (Hooker, 1904).

Because of the economic importance of *Q. saponaria*, Kew set up a programme aiming to introduce the tree ‘to India and to other British Possessions where it was likely to thrive’ (Hooker, 1904). Although the tree was found growing naturally only in the central part of Chile, it clearly had the capacity to grow in other areas. Hooker’s bulletin includes extracts of correspondence with aspiring growers, including these from Southern India:

‘Mr M. A. Lawson, F.L.S., gave the following account of plants raised on the Nilgiris in 1884: --.

*Q. saponaria*. A few only of the seeds of this Rosaceous plant which were sent from Kew have germinated. The plants, however, which have been raised are doing well.

In 1886, Mr. Lawson gave the following further information on the subject: --.

“*Q. saponaria*. – This plant thrives well in Ootacamund, and it is found that it can readily be propagated by means of cuttings, so that if it proves to be a tree of any value, it can be increased to any extent.”.

Since 1884, the trees on the Nilgiris have evidently done well. The following note shows that the bark of Indian-grown trees contains fully as much saponin as the bark imported into this country from South America: --

Mr. D. HOOPER, F.C.S., F.I.C., Quinologist to the Government of Madras, to ROYAL GARDENS, KEW.
The Laboratory, Oolacamund,
June 19, 1894.
DEAR MR. MORRIS,
You will be glad to know that the Quillaia Bark tree grows well here, and the bark of a ten-year-old tree contains as much saponin as the bark found in the London market. I do not know if the tree has been tried anywhere else in the East.
Yours sincerely, (Signed) D. HOOPER.
J. R. Jackson, Esq., A.L.S.,
Royal Gardens, Kew.’
DEAR SIR,
WITH reference to your enquiry respecting quillajaia bark, there is a good and increasing demand for this article; prices at this moment rule low, the present quotations ranging from £12 to £12 10s. per ton nett. With compliments,
I remain,
Yours faithfully,
H. ARNOLD.’

Despite interest in propagating *Q. saponaria* elsewhere, this endeavour seems to have died a death, given that there do not appear to be reports of present-day stands of cultivated *Q. saponaria* trees in Asia. This contrasts with the extensive effort invested in establishing plantations of a different South American tree cinchona (the fever tree), which produces the anti-malarial compound quinine, in India and Java in the late nineteenth century (Honigsbaum, 2001).

## 5 The Discovery Of Saponin Vaccine Adjuvants

A discovery in France in the 1920s would ultimately pave the way to a whole new commercial arena for the soapbark tree. At that time, French veterinarian Gaston Ramon was working on the development of effective vaccines for diphtheria and tetanus at the Pasteur Institute in Paris. Ramon realized that local inflammation at the inoculation site was associated with more effective vaccination (Ramon, 1926). He therefore carried out experiments in which he included various substances that were irritating to the tissues in his injections, including breadcrumbs, tapioca, starch, oil and ‘saponin’ (Ott & Van Nest, 2007; Warshakoon et al., 2009). These and subsequent experiments by others provided evidence that ‘saponin’ was an effective vaccine adjuvant in animals. The discovery of immune-boosting ‘irritant’ substances, which Ramon named ‘adjuvants’ (derived from the Latin meaning ‘to aid’) (Ramon, 1926), represented a major advance for the field of immunology. Ramon was nominated 155 times for the Nobel Prize in Physiology or Medicine between 1930 and 1953 for his many seminal contributions to vaccine development, but was never awarded the prize (Butler, 2016).

The literature on the sources of ‘saponin’ used in these early animal immunisation experiments in the 1920s and 30s is somewhat opaque. It is, in general, not clear what type of saponin was used or how pure it was. Kofler notes in 1927 that the saponin preparation most commonly used for scientific purposes was ‘Saponin purissimum albissimum’, supplied by Merck, which was extracted from soapwort (*Saponaria officinalis* L.) (Kofler, 1927). Other commercially available saponins at that time were from *Gypsophila paniculata* L. and *Smilax* species (sarsaparilla root) (Dalsgaard, 1970). Of note, a saponin preparation referred to as ‘saponium purissimum album’, presumably from soapwort, was used as an adjuvant in the anthrax vaccine ‘Carbozoo’, developed by Mazzuchi in 1929 (Mazzuchi, 1929; Troger, 1932). Espinet (1951) noted the efficacy of plant saponins to enhance the potency of foot and mouth disease vaccines (Espinet, 1951). Richou and colleagues have been attributed with the first use of saponins extracted from *Q. saponaria* as adjuvants in animal vaccines (Ragupathi et al., 2011; Richou et al., 1964), although the reason why *Q. saponaria* was selected is unclear. By 1970, it was evident that multiple commercial and privately supplied sources of saponin were being investigated for their potential as adjuvants in foot and mouth disease vaccines, although frustratingly little information about the plant sources is provided (Table 1) (Dalsgaard, 1970). Over the last ~60 years, the major focus on saponin immunostimulants for vaccine use has been on saponins extracted from *Q. saponaria* bark (Ragupathi et al., 2011).

**Figure 7:**
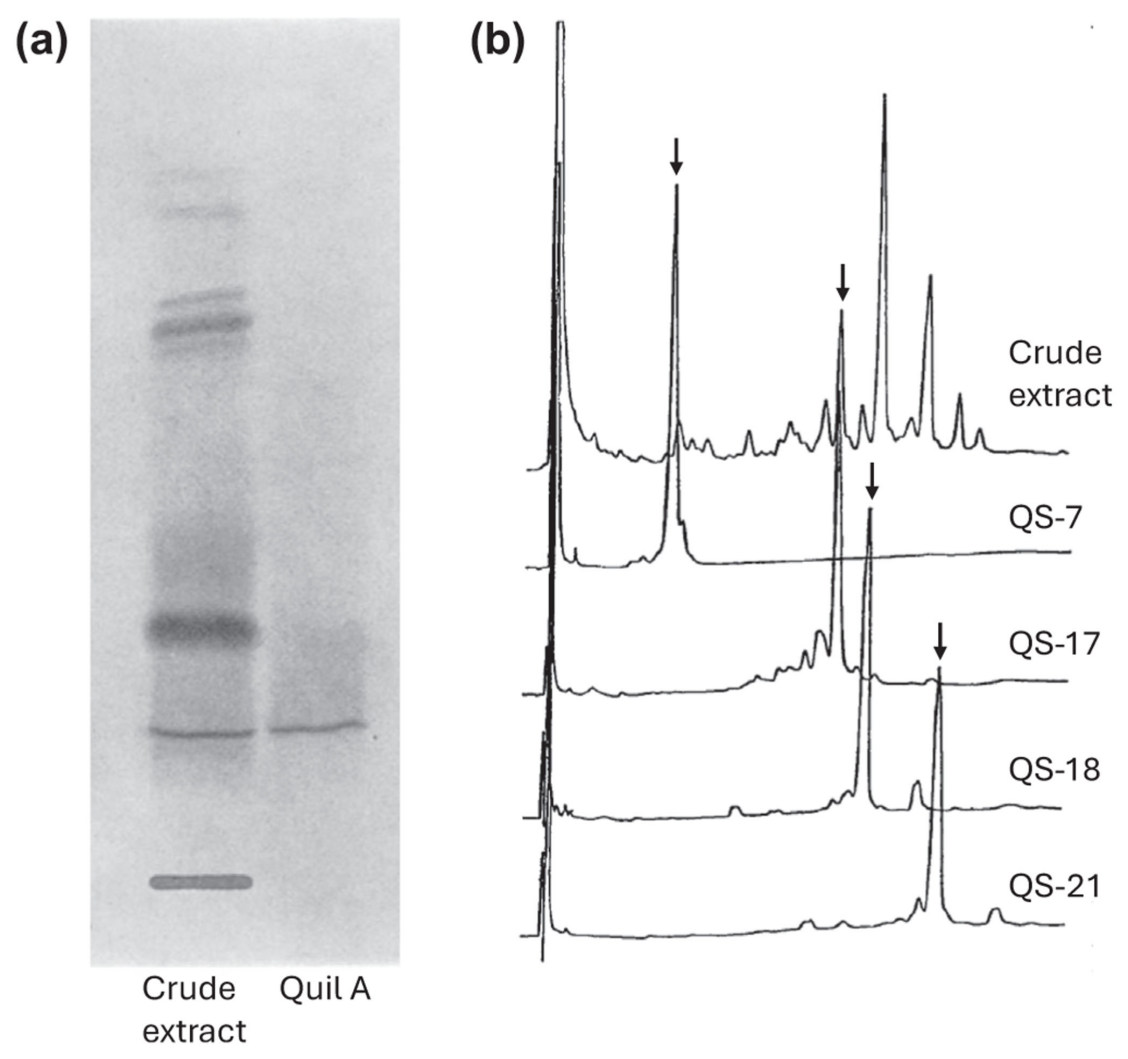
Fractionation of saponin adjuvant activity from crude bark extract. a) Thin layer chromatography of crude *Quillaja saponaria* bark extract and Quil A (from Dalsgaard, 1974); b) RP-HPLC traces of aqueous bark extract (top) and of fractions containing the four major adjuvant-active saponins QS-7, QS-17, QS-18 and QS-21 (from Kensil et al., 1991).

Crude saponin extracts are highly variable in composition and so are not a reliable source of adjuvant activity (Campbell & Peerbaye, 1992). In 1974, Dalsgaard partially purified the adjuvant activity of *Q. saponaria* bark extract using a combination of dialysis, ion exchange and gel filtration chromatography to yield a saponin fraction known as Quil A. Quil A had reduced toxicity and increased immunostimulant activity relative to crude bark extract (Dalsgaard, 1974) (Figure 7a). Although not suitable for human use because of a level of remaining residual toxicity (Sun et al., 2009), Quil A was subsequently widely exploited as an adjuvant in animal vaccines (Burakova et al., 2018; Cunha et al., 2012; Dalsgaard et al., 1977; Ren et al., 2014; Xiao et al., 2007). It was later shown that Quil A is a complex mixture containing four major adjuvant-active saponins that can be separated using reverse-phase high-performance chromatography (RP-HPLC) (Figure 7b) (Kensil et al., 1991). These saponin fractions were named QS-7, QS-17, QS-18 and QS-21 based on the peak number in the chromatography elution series. Of these, the most abundant component QS-18 was toxic to mice while QS-7 and QS-21 were less toxic. The fully elucidated structures of QS-7, QS-17, QS-18 and QS-21 are reported in Gui and Kenne (2000), Higuchi et al. (1988), Nyberg et al. (2000) and Guo et al. (2000), respectively. QS-7 is less abundant in bark extract than QS-21. Based on its potent adjuvant activity, low toxicity and reasonable abundance in bark extract, QS-21 was therefore taken forward for evaluation for use in humans (Kensil et al., 1995).

**Figure 8:**
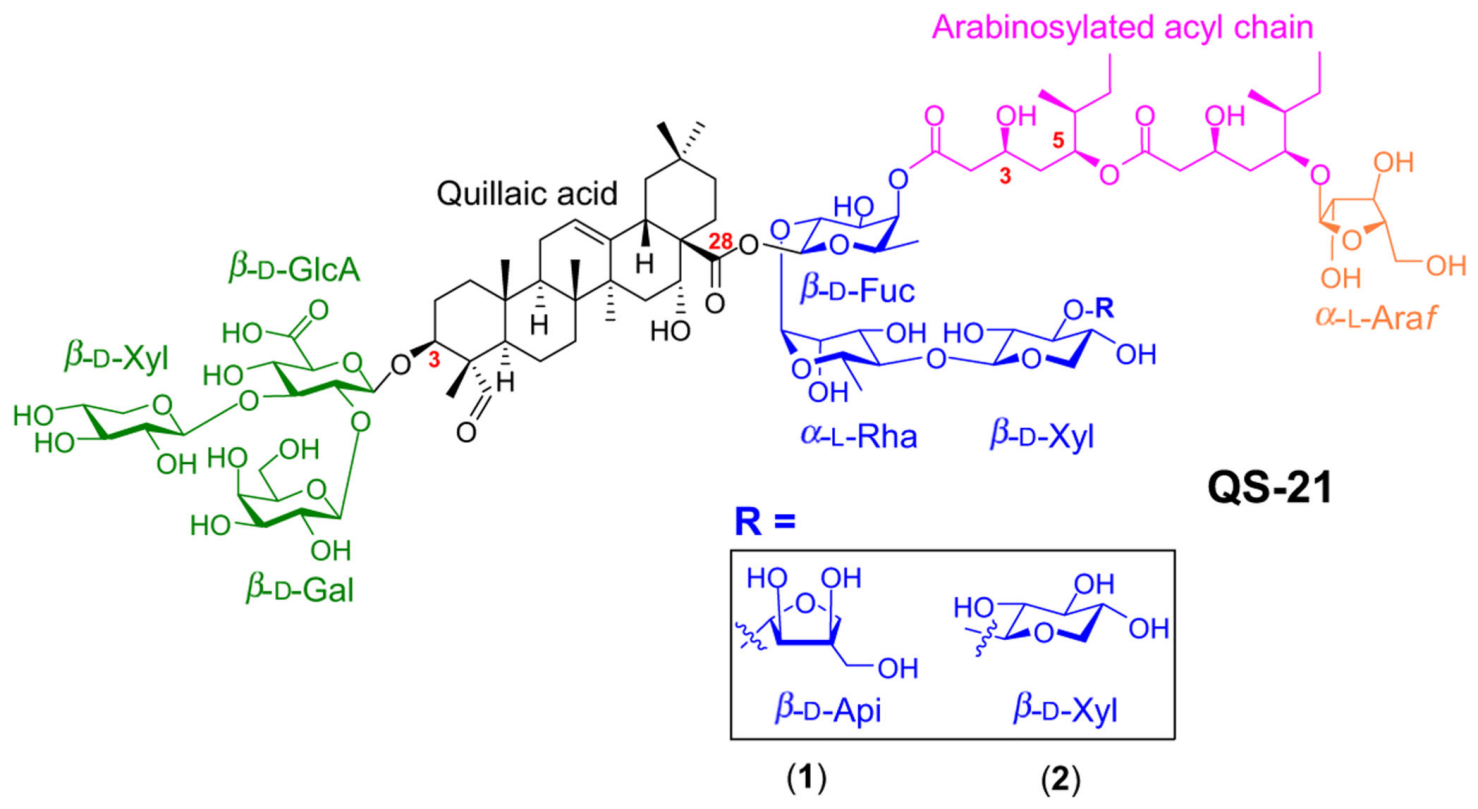
The structure of QS-21. QS-21 is a mixture of two structurally complex saponin isomers, D-apiofuranose (D-Api*f*) and D-xylopyranose (D-Xyl*p*). These compounds have a central core consisting of an oxygenated triterpenoid scaffold (quillaic acid) with a branched sugar chain at the carbon 3 (C3) position and a linear trisaccharide at the carbon 28 (C28) position. They differ only in the type of sugar that they have at the end of the tetrasaccharide chain.

QS-21 is in fact a mixture of two highly structurally complex saponin isomers, both having a central core consisting of an oxygenated triterpenoid scaffold (quillaic acid) with a branched sugar chain at the carbon 3 (C3) position and a linear trisaccharide at the carbon 28 (C28) position (Figure 8). In addition, they have a glycosylated C_18_ acyl chain linked to the saponin core via the initial sugar of the C28 sugar chain. The two saponins differ only in the type of sugar that they have at the end of the tetrasaccharide chain, this being either D-apiofuranose (D-Api*f*) or D-xylopyranose (D-Xyl*p*), the isomers occurring in the ratio 65%:35% (Kensil et al., 1991).

Human vaccines that consist of attenuated pathogens (e.g., the Sabin ‘live’ polio vaccine) or killed pathogens (e.g., the Salk inactivated polio vaccine) contain endogenous adjuvants. However, those that consist of purified antigens rather than intact microorganisms (e.g., the diphtheria-tetanus-pertussis vaccine and the hepatitis A and B vaccines) usually need to be supplemented with an exogenous adjuvant to increase the immune response (Marrack et al., 2009). The first adjuvants to be used in human vaccines were aluminium salts. The adjuvant activity of aluminium salts was discovered accidentally in 1926, when it was found that diphtheria toxin purified by the addition of alum was more immunogenic than the free toxin (Glenny et al., 1926). Aluminium salts have subsequently been used widely in human vaccines over the last hundred years (Marrack et al., 2009). In the 1990s, new adjuvants were developed to enhance immunity in vulnerable populations with poor responses to vaccination. These included Adjuvant Systems (AS), which consist of combinations of two or more immunomodulatory agents (Roman et al., 2024). The AS adjuvant system AS01 contains QS-21 along with another immunostimulant 3-*O-*desacyl-4^0^-monophosphoryl lipid A (MPL). AS01 was approved for human use in 2017 and is included in vaccines for malaria, shingles and respiratory syncytial virus, along with over 40 other vaccines currently in development. AS01-containing vaccines have now been administered to millions of individuals worldwide (Roman et al., 2024). Another adjuvant that contains *Q. saponaria* saponins has also been developed by the US company Novovax. This adjuvant, known as Matrix-M, consists of two different populations of nanoparticles containing different *Q. saponaria* saponin fractions (including both QS-21 and QS-7) mixed in a defined ratio and is used in vaccines for COVID-19 and malaria (Honigsbaum, 2024; Roman et al., 2024; Stertman et al., 2023).

## 6 Environmentally Sustainable Solutions to the Supply of *Q. saponaria* Saponins and Next-generation Vaccine Adjuvants

Demand for *Q. saponaria* (QS) saponins is growing as more QS saponin-containing vaccines are approved. This has led to concern about the robustness of the supply chain. It can take up to 25 years of growth before the bark of *Q. saponaria* trees is ready for harvesting. In some cases, the bark is stripped from the base of the trunk, causing the tree to wither and die. In other cases, loggers fell the whole tree and remove the bark, leaving the wood to rot in the forest. Extracting the bark is a laborious and expensive process, compounded by the fact that only around five out of every hundred *Q. saponaria* trees contain enough QS-21 to make the process commercially viable (Honigsbaum, 2024) (Figure 6). There are also concerns about the potential impact of natural disasters such as drought and wildfires on the *Q. saponaria* populations, most of which are wild trees.

The ability to biosynthesise QS-21 is restricted to *Q. saponaria* and the closely related species *Quillaja brasiliensis*. Complete chemical synthesis of the QS-21_Api_ and QS-21_Xyl_ isomers and QS-7 was achieved in the early 2000s but is challenging because of the structural complexity of the molecules (Deng, Adams, Damani, et al., 2008; Deng, Adams, & Gin, 2008; Kim et al., 2006; Wang et al., 2005). For example, synthesis of QS-21_Api_ required 76 steps, and the overall yield was negligible (Wang et al., 2005). Production of these molecules at a commercial scale by chemical synthesis is therefore not yet possible. In 2021, it was reported that natural forests were the principal source of 98% of *Q. saponaria* bark (Guerra & Sepúlveda, 2021). Desert King, one of the main suppliers of pharmaceutical-grade QS saponins, is currently working on selecting accessions of *Q. saponaria* with high levels of saponins and growing these in plantations in Chile, with the aim of trying to obtain sufficient yields from the branches and leaves of 5–10-year-old trees (Honigsbaum, 2024). QS saponins are also used for other purposes, for example, as emulsifiers and foaming agents in food and beverages, leading to the establishment of other efforts to extract saponin mixtures from young plantation-grown trees (San Martin & Briones, 1999; Schlotterbeck et al., 2015). Other strategies include agricultural plant microculture of both *Q. saponaria* and *Q. brasiliensis*, sapling microfarming, and establishment of plant cell cultures (Fleck et al., 2019; Guerra & Sepúlveda, 2021; Lv et al., 2024).

We recently sequenced the genome of *Q. saponaria* and elucidated the entire 20-step enzymatic pathway required for the biosynthesis of QS-21 (Martin et al., 2024; Reed et al., 2023). The accession that we sequenced (S10) was sourced from a UK nursery, which had previously obtained it from a Dutch supplier in 2013, a year before the Nagoya Protocol agreement entered into force, and so was available for us to use. We reconstituted the QS-21 pathway by transient *Agrobacterium*-mediated expression in tobacco, thereby demonstrating for the first time the production of QS-21 in a heterologous expression system (Martin et al., 2024). We also collaborated with the Keasling laboratory (University of California, Berkeley) to engineer the pathway into yeast. Yeast lacks many of the co-factors and precursors (e.g., UDP sugar donors) that are needed for the bioproduction of structurally complex, highly glycosylated plant compounds. Yeast engineering, therefore, required the introduction of a total of 18 auxiliary genes in addition to the 20 QS-21 pathway genes (i.e., 38 heterologous genes in total) and required multiple other modifications of the yeast genome (Liu et al., 2024). Substantial optimisation will be required to increase QS-21 yields in heterologous expression systems. However, these proofs of concept represent an important first step towards the bioproduction of QS saponins and the engineering of both known and novel new-to-nature saponin adjuvants in alternative platforms using engineering biology approaches (Spence et al., 2024).

The availability of the reference genome sequence for *Q. saponaria* (Reed et al., 2023) should also guide efforts to quantitatively and/or qualitatively modify the QS saponin content of the tree using either classical plant breeding or gene editing approaches.

## 7 Conclusion and Future Perspectives

QS-21 stimulates humoral and cell-mediated immunity against a wide range of antigens, and has advantages over aluminium salts by inducing a Th1-type immune response, necessary for controlling most intracellular pathogens (Laccaille-Dubois, 2019). Despite the success of QS-21, there is an urgent need for a deeper understanding of its mode of action (Laccaille-Dubois, 2019). In fact, QS-21 is not ideal as an adjuvant because it has haemolytic activity and is associated with pain at the injection site, limitations that are mitigated by formulation with a combination of cholesterol and phospholipid (Stertman et al., 2023). Efforts have been made to investigate the structural features of QS-21 that are important for immunostimulatory activity and toxicity using chemistry, but these have been limited because of the problems of selectively modifying such a structurally complex molecule (Fernández-Tejeda, 2017; Fernández-Tejeda et al., 2016). In fact, the soapbark tree makes not only QS-21 but also around a hundred other QS saponins, including QS-7. The sequenced *Q. saponaria* genome contains the genes encoding the enzymes needed to recapitulate this chemical repertoire (Martin et al., 2024; Reed et al., 2023). The ability to produce other saponins that are potent immunostimulants but have lower toxicity (such as QS-7 and other saponin analogues) in ‘free-from-tree’ heterologous expression platforms is now opening up unprecedented opportunities to carry out systematic investigation of the structure–activity relationships of these compounds. This enzyme toolkit could, in the future, be expanded to include enzymes from other plant species to generate even greater structural diversity, so enabling enzymes that carry out different types of modifications to be mixed and matched in order to design and make next-generation adjuvants for the vaccines of the future.

## Figures and Tables

**Table 1:** List of commercially available and privately supplied saponins used in a 1970 study by Dalsgaard.

Sample no	Origin
1	Saponin gereinigt MT, E. Merck, Darmstadt, lot no. 6272202
2	Saponin, The British Drug Houses Ltd., lot no. 3064490
3	Saponin “S”, Dr Th. Schuchardt, Munchen, lot no. not indicated
4	Saponin gereinigt, Riedel-de Haen, Seelze-Hannover, lot no. 59034 72
5	Saponin rein, Riedel-de Haen, Seelze-Hannover, lot no.59034 72
6	Saponina depurato, Carlo Erba, Milano, lot no. 70693 15,674
7	Saponin white, The British Drug Houses Ltd., lot no.2886790
8*	Saponin kindly supplied by l’Institut Francais de la Fievre Aphteuse, Lyon, France
9*	Saponin kindly supplied by Research Instute (Animal Virus Diseases), Pirbright, Great Britain
10	Saponin Weiss rein, E. Merck, Darmstadt, lot no. 5140023
11*	Saponin (Richou no. 2)
12*	Saponin (Richou no. 3)
13*	Saponin (Richou no. 4)
14*	Saponin (Richou no. 5)
15*	Saponin (Richou no. 7)
16*	Saponin (Richou no. 8)
“Q”	Aqueous extract of *Quillaja* bark prepared at the State Veterinary Institute for Virus Research, Lindholm, Kalvehave, Denmark

*Note*: Table adapted from Dalsgaard (1970).

* claimed to be *Quillaja saponaria* saponins. Samples 8 and 9 had previously been used as adjuvants in foot and mouth disease vaccines (according to personal communications referred to in Dalsgaard, 1970), while samples 11–16 had been shown to possess adjuvant activity in combination with staphylococcal anatoxin in rabbits (Kofler, 1927). The plant origins of the other purified saponin preparations obtained from commercial suppliers are not provided. Kofler notes in 1927 that the saponin preparation most commonly used for scientific purposes was ‘Saponin purissimum albissimum’, supplied by Merck, which was extracted from soapwort (*Saponaria officinalis*) (Kofler, 1927). It is not clear whether this corresponds to Sample 1. Other plant species used for industrial extraction of saponins at the time included *Gypsophila paniculata* and *Smilax* species (sarsaparilla root) (Dalsgaard, 1970). Thin-layer chromatography analysis carried out by Dalsgaard (1970) revealed that the profiles of samples 8, 9, 11, 14, 15 and 16 correlated well with the reference extract “Q”. However, samples 12 and 13 were different, sample 13 correlating more closely to samples 3 and 6.

## Data Availability

Data sharing not applicable to this article as no datasets were generated or analysed during the current study.

## Societal Impact Statement

Our current understanding of plants has been shaped by the entwining of different cultures. The Chilean soapbark tree, traditionally valued as a source of natural soap, was shown by serendipitous research in France in the 1900s to produce compounds that can boost the immune response to vaccines. One of these compounds, QS-21, was approved for use in human vaccines in 2017 and is now valued at >$100,000/g because of its growing importance as a vaccine adjuvant. This review tells the story of the humble soapbark tree and its elevation to fame as the source of one of the most sought-after naturally made immunostimulants.
